# Preoperative pain and achievement of the minimal clinically important difference for physical function one year after total hip arthroplasty: a prospective cohort study

**DOI:** 10.1007/s00402-026-06516-9

**Published:** 2026-10-13

**Authors:** Rubén Gajardo-Burgos, Ricardo Arteaga San Martín, Jorge Fuentes, Manuel Monrroy Uarac, Cristhian Saavedra-Santiesteban, Claudio Bascour-Sandoval, Eduardo Sáez de Villarreal

**Affiliations:** 1 Instituto de Ciencias del Movimiento y la Ocupación Humana, Universidad Austral de Chile, Valdivia, Chile; 2 Programa de Doctorado en Ciencias de la Actividad Física y del Deporte, Universidad Pablo de Olavide, Seville, Spain; 3 Clinical Research Lab, Department of Physical Therapy, Catholic University of the Maule, Talca, Chile; 4 Departamento de Ciencias de la Rehabilitación, Universidad de La Frontera, Temuco, Chile; 5 Faculty of Sport Sciences, Universidad Pablo de Olavide, Seville, Spain

**Keywords:** Total hip arthroplasty, Minimal clinically important difference, Preoperative pain, Functional recovery

## Abstract

**Purpose:**

To examine the association between preoperative social, clinical, and biological variables and the achievement of the minimal clinically important difference (MCID) on the WOMAC-function subscale one year post total hip arthroplasty (THA).

**Methods:**

In this prospective cohort study, 85 patients (aged ≥ 65) completed baseline preoperative assessment, and 53 completed the 12-month follow-up (32 lost to follow-up; 66.0% achieved MCID). The outcome was achieving WOMAC-function MCID (≥ 26.54 points). Multivariable logistic regression adjusted for baseline pain, age, sex, and BMI were performed. ROC curves evaluated discriminative capacity.

**Results:**

An exploratory ROC analysis identified a baseline pain threshold of 6.5 on the Numerical Rating Scale yielded an AUC of 0.740 (70% sensitivity, 77% specificity).

**Conclusions:**

Preoperative pain intensity independently associated with clinically meaningful functional improvement post-THA. An exploratory pain threshold of 6.5 on the NRS may provide an exploratory benchmark for future risk stratification.

## Introduction

Total hip arthroplasty (THA) is the gold standard surgical intervention for individuals with severe hip osteoarthritis who fail to obtain relief from conservative management. THA consistently yields substantial short- and medium-term improvements in pain, health-related quality of life, and overall functional capacity [1, 2]. Nevertheless, between 7% and 28% of patients report dissatisfaction with their postoperative outcomes [2, 3], spurring efforts to identify preoperative determinants of therapeutic success.

Several preoperative clinical, biological, and social variables have been associated with post-THA functional outcomes [4–8]. Consistently reported associated factors include baseline pain intensity [5], elevated body mass index (BMI) [5–7], presence of comorbidities [5], lower baseline functional capacity [5, 7], rurality [8] and educational level [5]. However, the extent to which these variables retain independent prognostic value when evaluated concurrently in adjusted multivariable models remains insufficiently clarified.

Although Patient-Reported Outcome Measures (PROMs), such as the Western Ontario and McMaster Universities Osteoarthritis Index (WOMAC) [9] are widely utilized to evaluate postoperative functional capacity [10–13], traditional analyses frequently focus on mean absolute score changes at the cohort level. In contrast, evaluating the minimal clinically important difference (MCID) provides an individual-level metric of perceived meaningful change, effectively distinguishing statistical significance from clinical relevance [14–16]. Crucially, achieving the MCID reflects relative functional gain conditioned by baseline severity rather than absolute postoperative performance [16]. In Spanish-speaking populations, an MCID threshold of 26.54 points (0–100 scale) has been validated for the WOMAC physical function subscale [17].

While recent investigations, such as that by Goh et al. [18], have explored factors influencing the achievement of the MCID following THA, these studies have predominantly focused on North American or European cohorts operating within high-income healthcare systems. Consequently, significant uncertainty remains regarding whether these prognostic models translate to older adults treated within public healthcare settings in Latin America [19], where specific socio-clinical determinants, such as rural residence, educational level, polypharmacy, and baseline functional dependence, may uniquely shape postoperative recovery [8, 19]. Evaluating these concurrent preoperative variables within an adjusted multivariable model in an underrepresented population is essential to enhance preoperative risk stratification and tailor clinical expectations.

Consequently, the objective of this study was to explore the association between preoperative social, clinical, and biological variables and the achievement of the MCID on the WOMAC-function subscale one year after THA in older adults from a Latin American public healthcare setting, using an adjusted multivariable model.

## Materials and methods

### Study design and participants

A prospective cohort study with a 12-month follow-up was conducted in accordance with the STROBE guidelines [20]. Ethical approval was granted by the XXXXX. All participants provided written informed consent.

Eligible participants were consecutive individuals aged ≥ 65 years with severe hip osteoarthritis awaiting primary THA between August 2017 and December 2018 at XXXXX. Exclusion criteria comprised severe cognitive impairment (Mini-Mental State Examination < 13) and uncompensated hearing loss precluding telephone assessments.

### Procedures and variables

Participants were recruited during their preoperative medical check-up. One researcher (Researcher one) conducted the informed consent process with potential participants, who confirmed their decision to participate by signing the informed consent form. Subsequently, the same researcher assessed cognitive capacity and gathered socio-biodemographic data.

A second researcher (Researcher two) contacted participants by telephone on the same day and twelve months later to administer the instruments measuring functional capacity and level of functional dependence. These instruments were administered via telephone, ensuring that only a single researcher performed all evaluations and guaranteed coverage of all individuals who attended the medical check-up, as they were contacted immediately after their consultation. This strategy was chosen because the study population is distributed across a large geographic area; thus, this approach permitted better access for participation and follow-up.

### Variables

**Explanatory variables**: Preoperative variables were analysed and grouped into three domains: (i) biological variables: age (years), weight (kg), height (m), body mass index (BMI), and sex (female/male); (ii) social variables: origin (rural/urban), educational level (low: primary education; high: secondary education or higher); and (iii) clinical variables: need for assistive devices (yes/no), current pain intensity (Numerical Rating Scale for pain), polypharmacy (use of two or more drugs: yes/no), functional dependence, and functional capacity.

**Outcome variable**: The primary outcome was the achievement of the MCID on the WOMAC physical function subscale at twelve months. For each participant, the change in the WOMAC-function score was calculated as the difference between the baseline value and the 12-month value (0–100 scale), where positive values indicate improvement. A participant was considered to have achieved the MCID when the change was ≥ 26.54 points, classifying the sample into “achieved MCID” and “did not achieve MCID” [17].

### Instruments

A structured interview was used to obtain the predictor variables, recording the results in an ad-hoc clinical file.

The level of functional dependence was measured using the Barthel Index, which evaluates activities of daily living (ADL) by determining the level of independence in their performance. This index consists of ten fundamental ADL items. Scores are assigned to each item, and a total score is calculated, allowing subjects to be classified as dependent (≤ 99 points) or independent (100 points). It has an administration time of approximately five minutes and demonstrates an internal consistency between 0.86 and 0.92. Furthermore, telephone administration of this instrument has shown a high degree of correspondence with face-to-face administration [21].

Pain intensity was assessed using the Numerical Rating Scale (NRS), given that it is a quick, easy-to-administer, highly reproducible instrument widely used in clinical practice. To evaluate pain, patients were asked to rate their pain while remaining still, where 0 represents “no pain” and 10 represents the “worst imaginable pain” [22].

Functional capacity was defined as the individual’s ability to decide and act in their activities of daily living [23]. For the evaluation of functional capacity, the WOMAC^®^ Index 3.1 was used, which is a widely utilized and validated instrument for measuring this variable in people with hip osteoarthritis [24]. It takes five minutes to administer and assess three dimensions (pain, joint stiffness, and physical function) using a battery of 24 questions, with a total score ranging from 0 to 96 points, where a higher score indicates poorer functional capacity. It has shown good internal consistency values. Moreover, it has shown good correspondence when administered via telephone [25]. The data for each dimension and the final score were standardized to a range of 0 to 100. To determine the cutoff point for the MCID, the values provided by Quintana et al. (2005) [17] for the Spanish version of the WOMAC physical function was used, establishing a cutoff point of 26.54 out of 100 for the WOMAC-function subdomain. Consequently, the population was divided into the group that “achieved MCID (≥ 26.54)” and the group that “did not achieve MCID (< 26.54)”. While MCIDs are recognized as context-dependent metrics influenced by baseline severity, instrument version, and estimation methods (anchor vs. distribution) [26], this established reference was selected to facilitate comparison with international literature, given the current lack of population-specific MCID thresholds for Chilean cohorts.

### Data analysis

JASP v0.19.x software was used for statistical analysis. A descriptive analysis was executed by calculating absolute and relative frequencies for qualitative variables. Quantitative variables were analyzed using the Shapiro-Wilk test to assess whether they conformed to a normal distribution. A non-normal distribution was confirmed; therefore, the median and interquartile range were used as descriptive measures. To evaluate potential follow-up attrition bias, baseline characteristics were compared between participants who completed the 12-month follow-up (*n* = 53) and those lost to follow-up (*n* = 32) using the Mann-Whitney U test for continuous variables and the Chi-square test (or Fisher’s exact test) for categorical variables. Furthermore, to assess the impact of missing outcome data on model stability, an extreme-case sensitivity analysis was performed for the total enrolled cohort (*n* = 85). Multivariable logistic regression models were re-estimated under two extreme assumptions: a worst-case scenario (assuming all dropouts failed to achieve MCID) and a best-case scenario (assuming all dropouts achieved MCID). To determine differences between variables according to the condition of achieving the WOMAC-function MCID among the analyzed participants, baseline characteristics were compared between study participants. To analyze the association between preoperative variables and the achievement of the MCID on the WOMAC-function subscale at twelve months, a multivariable binary logistic regression model was constructed. The variables included in the main model were selected a priori based on their clinical relevance and prior evidence, including age, sex, body mass index (BMI), and baseline pain level. Baseline WOMAC-function score was deliberately excluded from multivariable modeling to avoid mathematical coupling (part-whole correlation), as the baseline functional score is an intrinsic mathematical component of the outcome variable (MCID achievement: Δ WOMAC-function ≥ 26.54). This approach was used to control potential confounding factors and avoid model overfitting, in line with methodological recommendations for clinical prediction studies. As a secondary analysis, an alternative parsimonious model was constructed, including variables with a stronger association in the univariate analysis, to evaluate the robustness of the findings. To assess models’ stability and address the potential risk of overfitting and parameter imprecision inherent to the sample size, an internal validation using non-parametric bootstrapping with 1,000 resamples was conducted. Bias-Corrected and Accelerated (BCa) 95% confidence intervals were estimated for model parameters and Odds Ratios (ORs). Multicollinearity among predictors was assessed by calculating the Variance Inflation Factor (VIF). ORs with their 95% confidence intervals (95% CIs) were estimated. Finally, a receiver operating characteristic (ROC) curve analysis was performed to evaluate the discriminative capacity of baseline pain intensity. The area under the curve (AUC) was used to estimate overall diagnostic power, and the cutoff point was determined using the maximum Youden’s J statistic (Jmax). The level of significance used was *p* < 0.05.

## Results

**Fig. 1:**
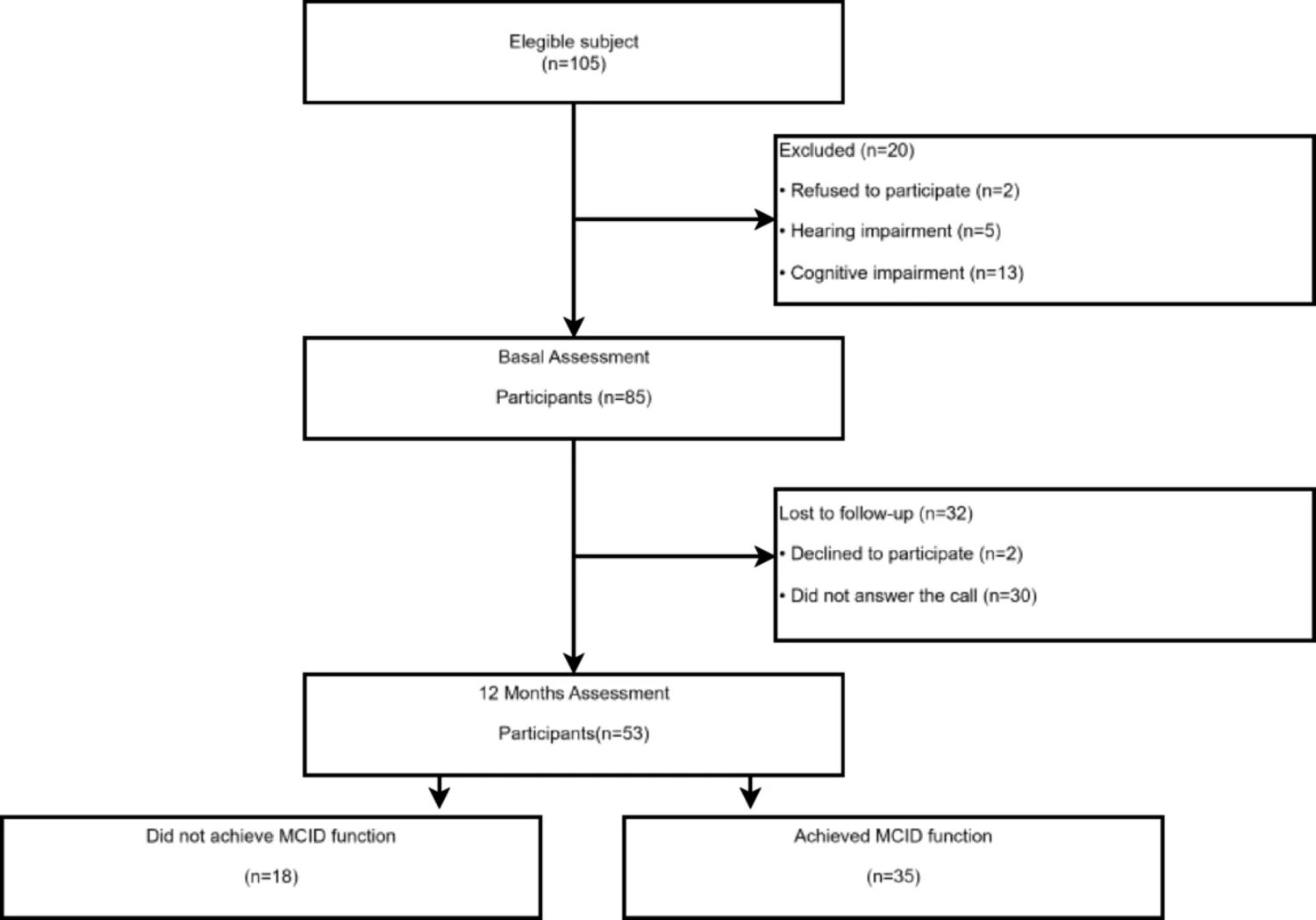
Flow diagram of participant recruitment, exclusion, follow-up, and WOMAC-function MCID classification at 12 months post-total hip arthroplasty

Of 85 eligible patients enrolled at baseline, 53 (62.4%) completed the 12-month follow-up (Fig. 1). Comparison between retained participants (*n* = 53) and dropouts (*n* = 32, 37.6%) showed no statistically significant differences in baseline characteristics (Table 1). However, given the limited sample size of dropouts, non-statistically significant comparisons do not rule out differential attrition bias. In the complete-case primary model (*n* = 53), 66.0% achieved the MCID. Sensitivity analysis across the entire enrolled cohort (*n* = 85) confirmed the robustness of baseline pain as a predictor: under the best-case scenario, baseline pain remained a strong independent predictor (OR = 1.418, 95% CI: 1.136–1.770, *p* = 0.002), matching the primary model effect size. Even under the highly conservative worst-case scenario, baseline pain maintained a positive trend toward significance (OR = 1.199, 95% CI: 0.995–1.445, *p* = 0.056). Overall cohort-wide MCID achievement rates bounded between 41.2% (worst case) and 78.8% (best case).

**Table 1:** Baseline characteristics of study participants and dropouts

	Participants *n* = 53 (62.36%)	Dropouts *n* = 32 (37.64%)	*p*-value
Biological			
Age	71 [67–73]	73.5 [68–77.25]	0.148
BMI	26.59 [23.92–31.63]	27.77 [25.85–29.12]	0.711
Sex			0.887
Women	29 (54.72)	17 (53.12)	
Men	24 (45.28)	15 (46.88)	
Social			
Rurality			0.126
Rural	9 (16.98)	10 (31.25)	
Urban	44 (83.02)	22 (68.75)	
Educational Level			0.635
Low	27 (50.94)	18 (56.25)	
High	26 (49.06)	14 (43.75)	
Clinical			
Pain (NRS)	7 [3.25–8.75]	7 [5–8]	0.563
Polypharmacy*			0.084
Yes	35 (67.31)	27 (84.38)	
No	17 (32.69)	5 (15.63)	
Assistive device			0.927
Yes	32 (60.38)	19 (59.38)	
No	21 (39.62)	13 (40.63)	
Independence			0.554
Yes	30 (56.6)	16 (50)	
No	23 (43.4)	16 (50)	

*Data available for 52 participants

*BMI *Body Mass Index*, NRS *Numerical Rating Scale,* IQR Interquartile Range*

Data are presented as n (%) representing within-group column percentages

Overall, 66.0% (*n* = 35) of participants achieved the WOMAC-function MCID at 12 months. Comparisons demonstrated that baseline pain intensity was significantly higher in patients who achieved the MCID (median 7 [IQR 5–9]) compared to those who did not (median 3 [IQR 1–6]; *p* = 0.005; Table 2). Rural residence was also associated with higher MCID achievement (100% vs. 59.1%, *p* = 0.018), though rural sample size was small (*n* = 9). Age, sex, BMI, education, polypharmacy, assistive device use, and baseline functional dependence showed no significant group differences (*p* > 0.05; Table 2).

**Table 2:** Preoperative comparison between participants who achieved and did not achieve the WOMAC-function MCID at 12 months

	Achieved WOMAC-function MCID n = 35 (66.04%)	Did not achieve WOMAC-function MCID n = 18 (33.96%)	p-value
Biological			
Age	71 [68–73]	69.5 [66.25–74.5]	0.651
BMI	27.34 [23.58–31.04]	26.58 [26.03–31.6]	0.610
Sex			0.930
Women	19 (54.29)	10 (55.56)	
Men	16 (45.71)	8 (44.44)	
Social			
Rurality			0.018
Rural	9 (25.71)	0 (0)	
Urban	26 (74.29)	18 (100)	
Educational Level			0.101
Low	15 (42.86)	12 (66.67)	
High	20 (57.14)	6 (33.33)	
Clinical			
Pain (NRS)	7 [5–9]	3 [1–6]	0.005
Polypharmacy*			0.053
Yes	26 (76.47)	9 (50)	
No	8 (23.53)	9 (50)	
Assistive device			0.089
Yes	24 (68.57)	8 (44.44)	
No	10 (31.43)	11 (55.56)	
Independence			0.289
Yes	18 (51.43)	12 (66.67)	
No	17 (48.57)	6 (33.33)	

*Statistically significant (p < 0.05). MCID: Minimal Clinically Important Difference (≥26.54 WOMAC points)

*BMI *Body Mass Index, *NRS* Numerical Rating Scale

Data are presented as n (%) representing within-group column percentages

In the primary multivariable model (Table 3), preoperative pain intensity was the sole independent predictor of achieving the MCID (OR = 1.42, 95% CI: 1.11–1.82, *p* = 0.006). Internal validation via 1,000 bootstrap resamples confirmed the stability and robustness of this finding, with baseline pain retaining a statistically significant association (Bootstrap OR = 1.474, 95% BCa CI: 1.058–2.204). Each 1-point increase in preoperative pain NRS was associated with a 42% increase in the odds of achieving clinically meaningful functional gain. Age (*p* = 0.902), male sex (*p* = 0.971), and BMI (*p* = 0.810) were not significant. The bootstrap procedure highlighted the parameter imprecision for secondary covariates in this sample, particularly for male sex (Bootstrap OR = 1.083, 95% BCa CI: 0.084–9.773). The model demonstrated moderate explanatory power (Nagelkerke R2 = 0.291, *p* = 0.038), acceptable discrimination (AUC = 0.757), and no multicollinearity (VIF ≤ 1.084).

**Table 3:** Binary logistic regression model evaluating clinical factors associated with MCID achievement

Variable	β (SE)	Odds ratio (OR)	95% CI OR	Wald	*p*-value	Bootstrap OR*	95% CI BCa (Bootstrap)*
Baseline pain (NRS)	0.35 (0.126)	1.42	1.108–1.818	7.706	0.006	1.474	1.058–2.204
Age (years)	−0.009 (0.07)	0.991	0.865–1.137	0.015	0.902	0.991	0.785–1.331
Sex (Male)	0.028 (0.764)	1.028	0.230–4.594	0.001	0.971	1.083	0.084–9.773
BMI	−0.018 (0.074)	0.982	0.849–1.136	0.058	0.810	0.978	0.813–1.183

Model summary: Nagelkerke R² = 0.291 (p = 0.038); AUC = 0.757; Max VIF = 1.084. *p < 0.05

*β* Unstandardized coefficient, *SE* Standard error, *OR* Odds ratio, 95% *CI* 95% confidence interval, *Wald* Wald statistic, *NRS N*umerical rating scale, *BMI* Body mass index, *BCa* Bias-Corrected and Accelerated

* Based on 1,000 successful non-parametric bootstrap replicates

In the secondary parsimonious model (Table 4) incorporating baseline pain, polypharmacy, and assistive device use, baseline pain remained the only significant predictor (OR = 1.30, 95% CI: 1.05–1.63, *p* = 0.019; Nagelkerke R2 = 0.324, AUC = 0.794). Internal validation via 1,000 bootstrap resamples confirmed the stability and robustness of this finding, with baseline pain retaining a statistically significant association (Bootstrap OR = 1.350, 95% BCa CI: 1.002–1.805). ROC analysis confirmed significant discriminative performance of preoperative pain (AUC = 0.740, 95% CI: 0.59–0.90, *p* = 0.005). The optimal preoperative pain cutoff identified by Youden’s J statistic (Jmax = 0.46) was 6.5 on the NRS, yielding 70.0% sensitivity and 77.0% specificity for predicting WOMAC-function MCID achievement.

**Table 4:** Binary logistic regression model evaluating factors associated with MCID achievement

Variable	β (SE)	Odds ratio (OR)	95% CI OR	Wald	*p*-value	Bootstrap OR*	95% CI BCa (Bootstrap)*
Baseline pain	0.265 (0.113)	1.303	1.045–1.625	5.518	0.019	1.350	1.002–1.805
Polypharmacy (Yes)	0.928 (0.710)	2.529	0.629–10.164	1.709	0.191	2.706	0.502–17.418
Assistive device (Yes)	1.172 (0.702)	3.230	0.815–12.796	2.785	0.095	3.509	0.531–17.324

Model summary: Nagelkerke R² = 0.324 (p = 0.004); AUC = 0.794; Max VIF = 1.023. *p < 0.05

*β* Unstandardized coefficient, *SE* Standard error, *OR* Odds ratio, 95% *CI* 95% confidence interval, *Wald* Wald statistic, *BCa* Bias-Corrected and Accelerated

* Based on 1,000 successful non-parametric bootstrap replicates

## Discussion

Our findings indicate that preoperative pain intensity is the main factor associated with achieving a clinically significant change in function from the patient’s perspective, even after adjusting for relevant clinical variables. Specifically, an exploratory cutoff point of 6.5 on the baseline pain NRS was identified, demonstrating acceptable discrimination for individuals with a higher probability of achieving an MCID in WOMAC-function.

A relevant finding of our study is that patients with higher baseline pain intensity had a greater probability of achieving the MCID. This finding aligns with the well-documented ‘room-for-improvement’ phenomenon. Patients presenting with greater baseline symptom severity start from a worse functional state [27], providing a larger capacity for gain and a higher likelihood of reaching an MCID [5, 16]. Previous literature suggests that THA is highly effective in cases of severe impairment, allowing more substantial changes in the patient’s perception of function, even if optimal absolute levels are not reached [5, 16]. Conversely, subjects with low baseline pain levels would obtain changes that are not considered relevant from the patient’s perspective. This could be because proximity to an optimal functional condition limits the magnitude of perceptible change following THA, as the MCID is conditioned by the baseline status [16, 18]. When interpreting functional recovery after hip surgery, a critical methodological distinction must be drawn between achieving a MCID and reaching a satisfactory absolute postoperative functional state [5, 16, 18]. MCID measures the magnitude of intra-individual change relative to baseline. Consequently, patients presenting with severe preoperative pain or lower baseline functional scores possess greater mathematical headroom to demonstrate substantial improvement [5, 16, 27]. As highlighted by Ramadanov et al. (2025) [28] in their evaluation of post-operative outcomes versus score differences in hip replacement, relying solely on change-based metrics without contextualizing absolute post-operative function can obscure whether a patient has achieved a truly acceptable clinical state [18, 28]. While change scores reflect relative surgical gain, absolute post-operative values determine actual functional capability and symptom resolution [12, 16, 18]. Therefore, future studies evaluating hip interventions should report both delta-based thresholds (MCID) and absolute functional targets to provide a comprehensive and unbiased assessment of clinical success [12, 18, 28].

Additionally, the ROC curve analysis identified a cutoff point of 6.5 on the preoperative pain scale as the optimal threshold to discriminate patients who will achieve clinically relevant improvement. This value is concordant with thresholds previously described in the literature to categorize severe pain in chronic musculoskeletal conditions [29] and in candidates for arthroplasty [30], suggesting the existence of a clinically significant pain range with prognostic implications. Likewise, the area under the curve (AUC = 0.74) indicates an acceptable discriminative capacity within this sample. However, because this cutoff was derived and evaluated within the same cohort (*n* = 53) without internal or external validation, these diagnostic metrics (sensitivity, specificity, AUC) are likely optimistic due to sample-specific overfitting. Consequently, this threshold must be framed strictly as exploratory rather than an established clinical stratification tool.

In contrast, no statistically significant associations were detected between achieving the MCID and secondary biological, clinical, or social variables, including age, sex, body mass index, polypharmacy, assistive device use, or educational level. Although some studies have reported associations between these variables and postoperative outcomes, the evidence is heterogeneous. On one hand, it has been reported that individuals with higher education present greater improvements in WOMAC pain and function [31, 32]; however, this was not found in our population. On the other hand, the relationship between BMI or sex and the improvement of functional capacity post-THA is controversial [33–37], indicating that these variables should not be considered a determining factor in post-THA outcomes. Another possible explanation is that the models used explain only a moderate proportion of the outcome variability, suggesting the existence of other unconsidered factors. In this regard, psychosocial variables such as pain catastrophizing [38], patient expectations, and emotional state [7] have shown a significant influence on functional outcomes after THA and could improve the predictive capacity of future models.

### Limitations

This study has several methodological limitations. First, the effective sample size at 12 months was small (*n* = 53), which constrained statistical power, widened confidence intervals for secondary covariates (e.g., sex), and increased the risk of model overfitting. Second, a high dropout rate was recorded (37.6%). Although there is no consensus in prospective observational studies, Grooten et al. (2019) [39] propose a response rate of 67%. Although baseline characteristics did not differ significantly between completers and dropouts, low statistical power precludes ruling out differential attrition bias. Extreme-case sensitivity analysis established that cohort-wide MCID achievement lies between 41.2% and 78.8%, though baseline pain retained its predictive directionality and statistical stability across both extreme outcome bounds. Third, baseline WOMAC-function was deliberately excluded from the multivariable regression to avoid mathematical coupling, meaning the independent prognostic value of baseline pain must be interpreted considering baseline functional severity. Fourth, the baseline pain cutoff of 6.5 was derived exploratory via ROC analysis within the same cohort without external validation, meaning sensitivity and specificity metrics may be optimistic. Fifth, although applying a fixed MCID threshold (26.54 points) offers a clear binary benchmark for clinical responsiveness, dichotomizing continuous change scores carries known methodological limitations, including reduced statistical power and arbitrary categorization near the cutoff. As recently emphasized by Ramadanov (2025) [26], MCID thresholds are highly context-dependent and vary across populations, baseline function, and calculation methodologies (anchor, distribution, or ROC-based approaches). Future studies should aim to validate Chilean-specific MCID cutoffs across diverse baseline severity strata to further refine patient-centered outcome evaluation. Finally, our model explains 29% of the variance in achieving the WOMAC-function MCID, implying that other variables explain the remainder. Furthermore, while internal validation via bootstrapping was performed, external validation in independent cohorts remains necessary.

Despite these limitations, this study highlights the prognostic relevance of baseline pain. While the identified pain threshold of 6.5 provides a preliminary, patient-centered benchmark, independent prospective validation is necessary before it can be applied as a decision-making tool in clinical practice.

## Conclusion

Preoperative pain intensity was independently associated with achieving a clinically significant functional improvement one year after total hip arthroplasty. Patients with higher baseline pain present a greater margin for improvement, with an exploratory NRS threshold of ≥ 6.5 indicating a higher likelihood of achieving the MCID, pending independent validation. Conversely, social, clinical, and biological variables such as rurality, educational level, polypharmacy, use of assistive devices, sex, age, or body mass index demonstrated no statistically significant independent association in multivariable models.

## Data Availability

No datasets were generated or analysed during the current study.
